# GPCRs as targets for flavonoids in cancer cells: new options for intervention

**DOI:** 10.37349/etat.2024.00268

**Published:** 2024-08-30

**Authors:** Katrin Sak

**Affiliations:** University of East Anglia, UK; NGO Praeventio, 50407 Tartu, Estonia

**Keywords:** Flavonoids, G protein-coupled receptors, cancer growth, metastasis

## Abstract

For a long time, the family of receptor tyrosine kinases, including epidermal growth factor receptor and insulin-like growth factor 1 receptor, was regarded as the main players stimulating cell proliferative signaling. Today, it is increasingly clear that many G protein-coupled receptors (GPCRs) are also involved in controlling the hallmarks of cancer by activating diverse intracellular signaling networks. GPCRs can therefore be considered as promising drug targets for fighting against diverse types of human malignancies. Although plant polyphenols, flavonoids, are well known for their diverse anticancer effects inhibiting the growth, proliferation, migration, and invasion of malignant cells, involvement of GPCRs in these activities has still remained largely unelucidated. Therefore, in this review article, the current knowledge about the role of GPCRs in anticancer action of structurally varied flavonoids is compiled, highlighting the ability of these natural polyphenols to modulate the expression levels of GPCRs but also suppress the action of endogenous ligands and downstream tumor-promoting events. These data show that targeting the respective GPCRs by specific flavonoids may open new perspectives in the therapeutic intervention in human malignancies.

## Introduction

The global incidence of cancer has increased each year and will be increasing further. While in 2022, there were close to 20 million new cancer cases worldwide, this number will estimably reach 35 million by 2050 [1]. Despite advances in early detection and the development of novel therapeutic regimens, recurrence and metastasis have remained the leading causes of death in cancer patients [2–4]. Tumor metastasis, the spread of malignant cells from a primary site to distant organs, is a result of several biological events and represents a highly organized process, posing the biggest challenge to cancer treatment [3, 5]. This complex process consists of cell adhesion to extracellular matrix (ECM), induction of cellular motility and invasiveness, accompanied by epithelial-mesenchymal transition (EMT) and degradation of ECM under the action of zinc-dependent endopeptidases, matrix metalloproteinase (MMP)-2 and MMP-9 [4–7]. Overexpression of these enzymes is therefore related to high potential of metastasis, representing poor prognostic factors for patients [6, 8]. The detailed understanding of molecular mechanisms of malignant progression and metastasis is crucial for identifying potential therapeutic agents to improve the overall survival of patients [9].

## The role of G protein-coupled receptors in cancer development and progression

G protein-coupled receptors (GPCRs) comprise the largest and most diverse family of cell surface receptors in eukaryotes [10, 11]. They transduce extracellular signals via G protein-dependent and -independent pathways to control a variety of cellular functions, such as neurotransmission, immune response, homeostasis, cellular mobility, growth, and survival. Dysregulation of GPCRs is involved in numerous human diseases including neurodegenerative diseases, cardiovascular diseases, diabetes but also malignant disorders [10–12]. Over the past decade, the association of GPCRs with cancer has become more and more apparent and understanding their precise role in the initiation and development of cancer can open new perspectives to target these membranous receptors in the cancer treatment [10, 12].

Increasing evidence has indicated that aberrant expression of GPCRs and activating mutations in GPCRs can lead to ligand-independent constitutive pathways stimulating tumor progression and determining poor prognosis [10, 12]. Mutated GPCR genes have been indeed identified in more than a fifth of studied malignancies, with the highest mutation rate in gastrointestinal adenocarcinomas [10]. In addition, GPCR ligands are often overproduced during oncogenesis, in turn inducing excessive activation of their receptors and stimulating oncogenic signaling networks [10]. Moreover, GPCRs can contribute also to the establishment of a microenvironment that further promotes tumor formation and growth [12].

In general, GPCRs couple to heterotrimeric G proteins and are classified based on their ability to preferentially activate an α subunit of four subfamilies: Gαs, Gαi/0, Gαq/11, and Gα12/13 [10–12]. Through G proteins, GPCRs modulate numerous downstream signaling pathways that can importantly contribute to the tumor development, malignant growth, and metastasis [10, 12]. Gαs-coupled GPCRs stimulate adenylyl cyclase converting ATP into cyclic adenosine monophosphate (cAMP), whereas Gαi-coupled GPCRs block adenylyl cyclase and the formation of cAMP. The produced cAMP further activates protein kinase A (PKA) that can modulate several transcription factors [10]. Gαq-coupled GPCRs activate phospholipase C leading to an increase in inositol triphosphate levels that, in turn, is responsible for the rise of intracellular Ca^2+^ concentrations and regulation of various cellular responses [10]. Gα12/13-coupled GPCRs act via Rho-activating signaling cascades including mitogen-activated protein kinases (MAPKs) and Rho-associated protein kinases (ROCKs), resulting in changes in cytoskeleton [10]. In this way, the regulated downstream signaling pathways may essentially affect the course of carcinogenesis and malignant spread.

## Flavonoids as potential leads for novel anticancer agents

**Figure 1:**
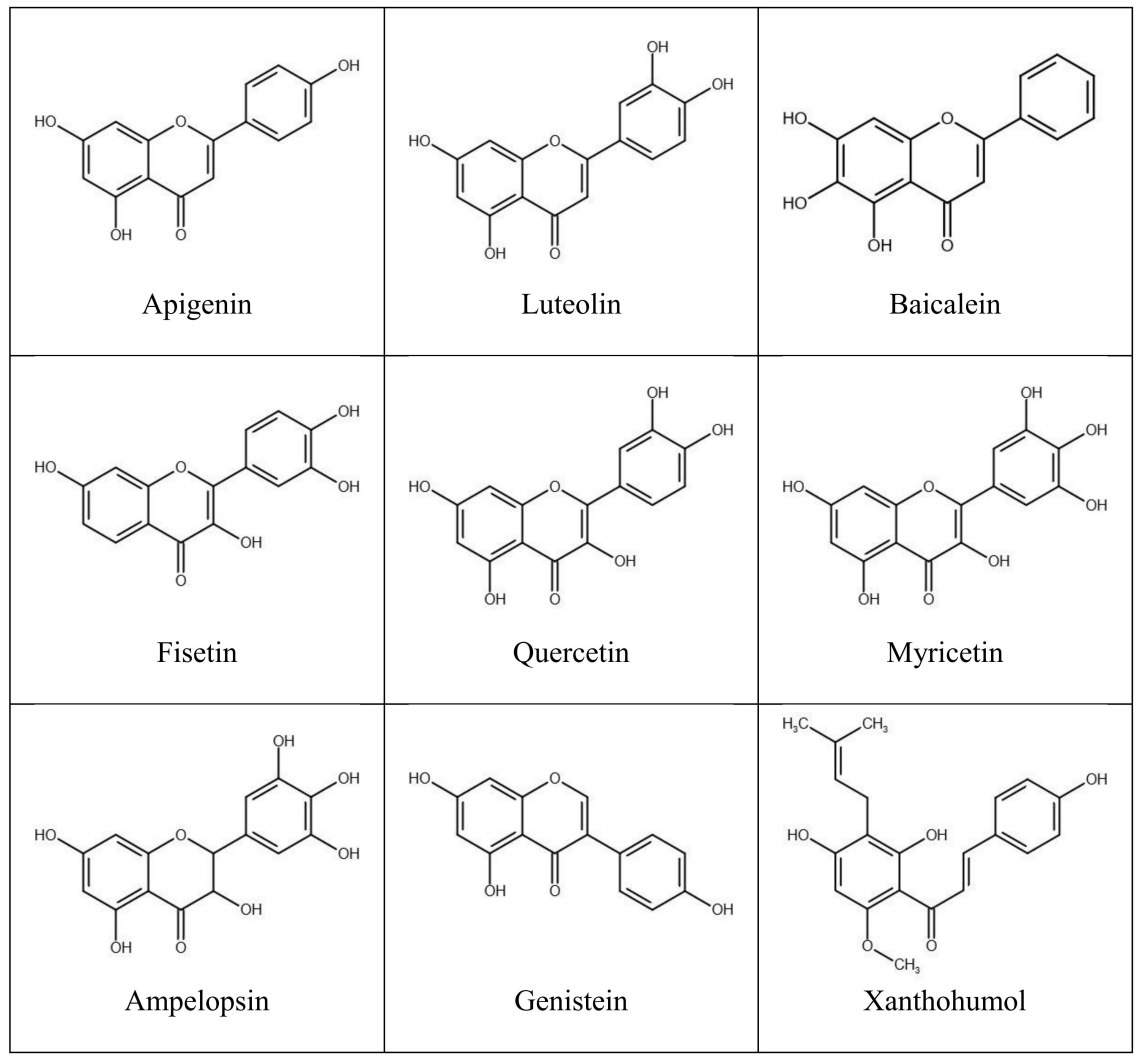
Structure of flavonoids which act on GPCRs in cancer cells

Over the past few decades, an increasing number of studies have demonstrated the anticancer role of plant-derived low-molecular weight polyphenolic compounds, flavonoids, in diverse types of malignancies. Flavonoids constitute the largest class of plant secondary metabolites, with the structure consisting of two benzene rings connected by a heterocyclic ring of three carbon atoms (C6-C3-C6) [11] (Figure 1). Based on their structural peculiarities, especially the presence of a double bond and a ketone group in the heterocyclic ring but also the number and pattern of hydroxyl moieties in flavan backbone, flavonoids can be divided into several subgroups, such as flavanols or catechins, flavones, flavonols, flavanones, isoflavones and anthocyanidins, besides chalcones with an opened central ring [11, 13]. Flavonoids are widely distributed in the plant kingdom, occurring naturally mostly in the form of glycosides [11, 13]. In this way, flavonoids can be abundantly found in human daily diet like fruits, vegetables, grains, legumes, spices, nuts, cocoa, tea, juices, and medicinal herbs [8, 11]. The consumption of flavonoids is usually about 1 g/day but can vary largely depending on cultural and regional habits [13]. In plants, flavonoids have several important functions, contributing to protection against biotic and abiotic stressors, pigmentation, pollinator attraction and seed germination [11, 13]. In humans, flavonoid glycosides undergo rapid hydrolysis to their aglycone forms being further converted into different conjugated metabolites which enter the systemic circulation, present in low micromolar concentrations [8, 14].

In recent years, increasing experimental evidence has shown various biological activities of flavonoids, such as antioxidant, anti-inflammatory, antimicrobial, antithrombotic, hypolipidemic, hypoglycemic, antiallergic and anticancer properties [11, 13]. Therefore, these plant secondary metabolites may be protective against several chronic diseases like cardiovascular disorders, neurodegenerative diseases, diabetes, and cancer [11, 13]. In malignant disorders, flavonoids can interfere with different cancerous processes, including proliferation, angiogenesis, and metastasis, inducing cell cycle arrest, promoting apoptosis, and restricting invasiveness through regulating diverse intracellular signaling pathways [8, 13, 14]. Several epidemiological studies have indeed revealed an inverse association between a higher intake of flavonoids and the risk of certain types of malignancies, especially those of epithelial origin [14–16]. However, the specific cellular targets and precise molecular mechanisms underlying the inhibitory action of flavonoids in cancer development and progression have still remained rather elusive [7, 15–18].

## The involvement of GPCRs in anticancer activities of flavonoids

Besides the other mechanisms, emerging experimental studies have demonstrated that flavonoids can exert their anticancer activities also through acting on different types of GPCRs [11]. These effects can be achieved by either modulating the expression levels of GPCRs in cell membrane or behaving as competitive antagonists impeding the binding of endogenous ligands to their receptors and inhibiting the subsequent tumor-promoting activities [11]. Most GPCRs, when overexpressed, can induce tumorigenic activities; however, some of them exhibit also anticancer effects [10, 12]. Therefore, the ability of flavonoids to decrease or increase the expression of these GPCRs may be crucial in interfering with malignant progression. In addition, flavonoids can modulate also the activity of GPCRs by acting as allosteric ligands [11]. In all these processes, chemical entities and structural peculiarities of flavonoids may play a key role in their ultimate biological effects [11].

In the following three subsections, the currently known data about the anticancer role of flavonoids mediated via regulating different types of GPCRs are discussed. For covering all the subject, a systematic search for articles was performed by the words “flavonoid” and “cancer” and all individual members of GPCRs listed in the IUPHAR/BPS Guide to Pharmacology (excluding those belonging to the subclass of orphan and other 7TM receptors) [19]. To the best of the author’s knowledge, this is the first review where G protein-coupled membranous receptors are considered as molecular targets for anticancer activities of flavonoids.

### Decrease in GPCRs expression by anticancer flavonoids

#### C-X-C chemokine receptor type 4 (CXCR4)

CXCR4 plays a critical role in the metastatic spread of different types of tumor cells mediating their homing to organs which express the specific ligand, C-X-C motif chemokine ligand 12 (CXCL12) [3, 4, 7]. Besides inducing adhesion and invasion, CXCR4 can promote also the growth and survival of malignant cells in distant sites [3, 4, 7]. Overexpression of CXCR4 has been indeed detected in various human malignancies, including colon cancer, pancreatic cancer, breast cancer, ovarian cancer, and prostate cancer, presenting as an important pro-invasive factor that is associated with increased metastasis, resistance to conventional therapies, recurrence, and poor prognosis in patients [3, 4]. The expression of CXCR4 in normal healthy tissues is contrarily low or absent [3]. Therefore, the identification of CXCR4 inhibitors has a great potential to abrogate tumor metastasis [3, 4, 20].

The flavone apigenin (Figure 1) was recently shown to downregulate the expression of CXCR4 in a wide variety of human malignant cell lines, including those derived from colon and prostate cancers. In arsenic-transformed Beas-2B human bronchial epithelial cells (B-AsT), this flavone suppressed CXCR4 expression through the inhibition of nuclear factor-κB (NF-κB) transcriptional activity, resulting in the abolishment of constitutive and CXCL12-stimulated migration and invasion. Moreover, apigenin suppressed the expression of CXCR4 also in a xenograft mouse model, accompanied by the restriction of tumor growth [4]. Another natural flavone luteolin (Figure 1) reduced the expression of CXCR4 in human breast cancer cells MDA-MB-231, being associated with the suppression of cellular invasion. Luteolin could inhibit the metastasis also in B16F10 melanoma cells-bearing mice, decreasing the levels of CXCR4 in lung tissues [21]. The flavonol fisetin (Figure 1) exerted its anticancer activities in human non-small cell lung cancer cells A549 by reducing the expression of several tumor-promoting genes, including CXCR4 [17]. The flavonol myricetin (Figure 1) could suppress the CXCL12-CXCR4 axis in human prostate cancer cells PC-3 via inhibiting the activity of Moloney murine leukemia virus 1 (PIM1) and disrupting its interaction with CXCR4, leading to a significant decrease in CXCL12-induced cell migration. PIM1, a serine/threonine protein kinase, can directly bind to CXCR4 and facilitate its membranous expression, whereas the elevated co-expression of these two proteins, PIM1 and CXCR4, was correlated with aggressiveness and poor prognosis in prostate cancer patients. Myricetin was able to downregulate CXCR4 expression also in mouse models, restricting the lung and bone metastasis in PC-3 cells-implanted mice [7]. A decrease in the migratory and invasive potential of these androgen-independent prostate cancer cells was achieved also using the flavanonol dihydromyricetin or ampelopsin (Figure 1) via downregulation of the CXCR4 protein expression. In addition, ampelopsin restricted the lymph node and lung metastasis in orthotopic PC-3 tumor model in mice, associated with the suppression of CXCR4 levels [19]. Furthermore, the hops-derived prenylated chalcone xanthohumol (Figure 1) could repress the expression of CXCR4 in various cancer cell types, including human breast cancer cells MDA-MB-231 and colon cancer cells SW620, occurring at the transcriptional level and leading to the abrogation of CXCL12-induced invasion [3] (Table 1).

**Table 1:** Anticancer action of flavonoids mediated by different GPCRs

**Subfamily of GPCRs**	**GPCR**	**Flavonoid**	**Cancer model**	**Molecular mechanisms of biological effects**	**Reference**
Adrenoceptors	α_2C_-AR	Quercetin (0.1 µM)	MCF-10A human mammary epithelial cells	[^3^H]-NA binding to α_2C_-AR↓ 4-OHE_2_- and NA-induced γ-H2AX and apurinic sites↓	[14]
		Quercetin-3-*O*-glucuronide (0.1 µM)	MCF-10A human mammary epithelial cells	[^3^H]-NA binding to α_2C_-AR↓ 4-OHE_2_- and NA-induced γ-H2AX and apurinic sites↓	[14]
	β_2_-AR	Quercetin-3-*O*-glucuronide (0.1 µM)	MDA-MB-231 human breast adenocarcinoma cells	[^3^H]-NA to β_2_-AR↓ NA-induced ROS/NF-κB↓, cAMP/PKA↓, MAPK↓, MMP-9↓ NA-induced invasion↓	[8]
Cannabinoid receptor	CB_1_R	Quercetin (50 µM)	Caco2 human colon adenocarcinoma cells	CB_1_R mRNA and protein expression↑ PI3K/Akt/mTOR↓, JNK/JUN↑ Cell growth↓, cell cycle progression from S to G2/M↓, apoptosis↑, cell migration↓	[15]
		Quercetin (50 µM)	DLD-1 human colon adenocarcinoma cells	CB_1_R mRNA and protein expression↑ Cell growth↓	[15]
		Quercetin (0.5% of standard diet)	Intestinal tissue of AOM-treated C57BL/6J male mice	CB_1_R mRNA and protein expression↑ STAT3↓, p-STAT3↓, Bax/Bcl-2↑	[22]
Chemokine receptor	CXCR4	Apigenin (20 µM, 40 µM)	Arsenic-transformed Beas-2B human bronchial epithelial cells (B-AsT)	CXCR4 protein expression↓ Constitutive and CXCL12-induced cell migration and invasion↓	[4]
		Apigenin (40 mg/kg)	B-AsT cells-injected male athymic nude mice	CXCR4 protein expression↓ Tumor growth↓	[4]
		Luteolin (10 µM, 30 µM)	MDA-MB-231 human breast carcinoma cells	CXCR4 mRNA and protein expression↓ Cell invasion↓	[21]
		Luteolin (40 mg/kg)	Lung tissue of B16F10 murine melanoma cells-injected C57BL/6 male mice	CXCR4 mRNA and protein expression↓ The number of lung metastases↓	[21]
		Fisetin (40 µM)	A549 human non-small cell lung cancer cells	CXCR4 mRNA expression↓ Apoptosis↑	[17]
		Myricetin (25 µM, 50 µM)	PC-3 human prostate cancer cells	Disruption of PIM1/CXCR4 interaction↑, CXCL12-CXCR4 axis↓ CXCL12-induced migration↓	[7]
		Myricetin (25 mg/kg)	PC-3 cells-injected athymic nude mice	Phosphorylated CXCR4 expression↓ Lung and bone metastasis↓	[7]
		Ampelopsin (25 µM, 50 µM)	PC-3 human androgen-independent prostate cancer cells	CXCR4 protein expression↓ Cell migration↓, cell invasion↓	[20]
		Ampelopsin (300 mg/kg)	Orthotopic PC-3 tumor model in male severe combined immunodeficient mice	CXCR4 protein expression↓ Lymph node metastases↓, lung metastases↓	[20]
		Xanthohumol (50 µM)	MDA-MB-231 human breast adenocarcinoma cells	CXCR4 mRNA and protein expression↓ CXCL12-induced invasion↓	[3]
		Xanthohumol (50 µM)	SW620 human colon adenocarcinoma cells	CXCR4 mRNA and protein expression↓ CXCL12-induced invasion↓	[3]
Membrane estrogen receptor	GPR30	Baicalein (8 µM)	MCF-10A and MCF-12A human mammary non-tumorigenic epithelial cells	E2-induced GPR30-mediated SRC, EGFR, ERK1/2 and Akt signaling↓ Tumorigenesis↓	[16]
		Baicalein (10 µM, 15 µM)	MCF-7 human breast cancer cells	E2-induced GPR30-mediated EGFR, ERK1/2 and Akt signaling↓, MMP-9 mRNA and protein expression and activity↓ E2-induced GPR30-mediated cell migration, adhesion, and invasion↓	[5, 6]
		Baicalein (10 µM, 15 µM)	SK-BR-3 human breast cancer cells	E2-induced GPR30-mediated EGFR, ERK1/2 and Akt signaling↓ E2-induced GPR30-mediated cell migration, adhesion, and invasion↓	[5]
Parathyroid HR	PTHR1	Quercetin (80 µM, 100 µM)	U2OS and Saos-2 human metastatic osteosarcoma cells	PTHR1 mRNA and protein expression↓ Cell proliferation↓, migration↓, adhesion↓, invasion↓	[9]
Prostanoid receptor	EP3R	Genistein (50 µM, 80 µM)	Human oral (MMel-1, MMel-2), uveal (OCM-1, OCM-3) and cutaneous (G361, WM-115) melanoma cells	EP3R mRNA expression↓ PGE2-induced and EP3R-mediated IL-8 mRNA expression↓ Cell proliferation↓	[18]

4-OHE_2_: 4-hydroxyestradiol; Akt: protein kinase B; AOM: azoxymethane; AR: adrenoceptor; Bax: Bcl-2-associated X protein; Bcl-2: B cell lymphoma-2; cAMP: cyclic adenosine monophosphate; CB_1_R: cannabinoid receptor 1; CXCL12: C-X-C motif chemokine ligand 12; CXCR4: C-X-C chemokine receptor type 4; E2: 17β-estradiol; EGFR: epidermal growth factor receptor; EP3R: prostaglandin E receptor 3; ERK1/2: extracellular signal-regulated kinases 1/2; GPCR: G protein-coupled receptor; HR: hormone receptor; IL-8: interleukin-8; JNK: c-Jun N-terminal kinase; MAPK: mitogen-activated protein kinase; MMP-9: matrix metalloproteinase-9; mTOR: mammalian target of rapamycin; NA: noradrenaline; NF-κB: nuclear factor-κB; PGE2: prostaglandin E2; PI3K: phosphoinositide 3-kinase; PIM1: Moloney murine leukemia virus 1; PKA: protein kinase A; PTHR1: parathyroid hormone receptor 1; ROS: reactive oxygen species; SRC: proto-oncogene tyrosine-protein kinase Src; STAT3: signal transducer and activator of transcription 3

Therefore, certain flavonoids may be considered as novel efficient inhibitors of CXCR4 expression, holding a great potential to suppress the metastatic spread and progression of diverse types of human malignancies.

#### Parathyroid hormone receptor 1 (PTHR1)

PTHR1 is a GPCR that is widely expressed in metastatic tissues and cells, playing a crucial role in the pathophysiology of osteosarcoma [9]. The binding of parathyroid hormone (PTH) and PTH-related peptide (PTHrP) to this receptor has been shown to enhance the cell proliferation, migration, and invasion [9].

It was recently demonstrated that the flavonol quercetin (Figure 1) could significantly reduce the mRNA and protein expression of PTHR1 in human osteosarcoma cells U2OS and Saos-2, resulting in the suppression of proliferation, migration, adhesion, and invasion. Moreover, the knockdown of PTHR1 further enhanced the quercetin-induced anticancer effects [9] (Table 1).

#### Prostaglandin E receptor 3 (EP3R)

EP3R is a prostanoid receptor activated by its endogenous ligand prostaglandin E2 (PGE2). Like other PGE2-specific prostanoid receptors (EP1R, EP2R, EP4R), EP3R is implicated in angiogenesis, reduced host immunity, enhanced invasion and metastasis in cancer [18].

The soy isoflavone genistein (Figure 1) was shown to downregulate the EP3R expression leading to the inhibition of PGE2-induced and EP3R-mediated levels of interleukin-8 (IL-8) in diverse oral, uveal and cutaneous melanoma cell lines. PGE2, produced either by melanoma cells or tumor-surrounding inflammatory cells, can act as a promoter of tumorigenesis, with its elevated production, presenting an unfavorable prognostic factor for patients. The upregulated synthesis of IL-8 can, in turn, stimulate the malignant development and progression. Therefore, the ability of genistein to suppress the EP3R expression and decrease the IL-8 induction was associated with its growth inhibitory effects, providing a complementary weapon in the fight against melanoma [18] (Table 1).

### Increase in GPCRs expression by anticancer flavonoids

#### Cannabinoid receptor 1 (CB_1_R)

CB_1_R, a GPCR activated by cannabinoids, is mostly expressed in several brain regions but also in some peripheral areas such as gastrointestinal tract and smooth muscles, where it performs a number of activities [15, 22]. Several studies have described the anticancer action of cannabinoid receptor agonists, activating CB_1_R and controlling the growth and progression of tumoral cells [15, 22]. In addition, the overexpression of CB_1_R has been proposed as a favorable prognostic factor for different human malignancies [15].

The flavonol quercetin (Figure 1) was recently demonstrated to be able to significantly upregulate the CB_1_R expression in human colon adenocarcinoma cells, leading to the inhibition of downstream survival signaling of phosphoinositide 3-kinase (PI3K)/protein kinase B (Akt)/mammalian target of rapamycin (mTOR) and induction of the proapoptotic pathway of c-Jun N-terminal kinase (JNK)/JUN. These effects were associated with a decrease in cell growth, promotion of apoptosis and suppression of cell migration. Moreover, the anticancer action of quercetin was reinforced by the addition of an endogenous agonist of CB_1_R, anandamide, and counteracted by a specific CB_1_R antagonist, SR141716, proving the involvement of CB_1_R [15]. In addition, quercetin could increase also the expression of CB_1_R in a mouse model of induced colon cancer, inhibiting the mediators of proliferation (STAT3, p-STAT3) and inducing the markers of apoptosis [Bcl-2-associated X protein (Bax)/B cell lymphoma-2 (Bcl-2)], thereby leading to the suppression of colon carcinogenesis [22] (Table 1). These findings are of great interest for further development of novel agents for colon cancer therapy.

### Inhibition of ligand binding to GPCRs by anticancer flavonoids

#### Adrenoceptors (ARs)

Numerous studies have shown that chronic stress can trigger carcinogenesis, via the release of endogenous catecholamines such as adrenaline and noradrenaline (NA) from the adrenal gland and sympathetic nervous system [8, 11, 14]. The activation of cognate receptors, ARs α_1_, α_2_, β_1_, β_2_ and β_3_, by these neurotransmitters can promote the process of malignant progression and metastasis, inducing cell migration, invasion, angiogenesis, extravasation, and colonization [8, 11, 14]. Therefore, targeting neuroendocrine dynamics in tumor microenvironment may present a new therapeutic option for the management of stress-driven cancerous disorders [11].

In human mammary epithelial cells MCF-10A, the flavonol quercetin (Figure 1) and its major circulating metabolite quercetin-3-*O*-glucuronide could behave as antagonists for α_2C_-AR subtype, inhibiting the binding of [^3^H]-NA to this receptor, and leading to the prevention of NA-induced phosphorylation of histone H2AX (γ-H2AX) and premutagenic apurinic sites in DNA in the presence of 4-hydroxyestradiol (4-OHE_2_). These two flavonols may therefore be considered as chemopreventive agents for stress-related breast tumors by maintaining genome integrity [14]. Moreover, quercetin-3-*O*-glucuronide could act also as a competitive antagonist for β_2_-AR, inhibiting the binding of [^3^H]-NA to β_2_-AR in human breast adenocarcinoma cells MDA-MB-231. This resulted in the suppression of NA-induced reactive oxygen species (ROS), cAMP, MAPKs and NF-κB, with a decrease in the activity of downstream products such as MMP-9, eventually leading to the inhibition of NA-promoted invasion of malignant cells [8] (Table 1). Quercetin-3-*O*-glucuronide can therefore control both initiation as well as progression of breast cancer via interfering with the adrenergic signaling, providing a novel promising agent for combating stress-induced malignancies.

#### G protein-coupled estrogen receptor (GPR30)

GPR30 is a membranous estrogen receptor that functions independently of the classical nuclear estrogen receptors, ERα and ERβ, and mediates both rapid non-genomic signaling via multiple intracellular pathways as well as transcriptional events [5, 6, 13, 16]. The overexpression of GPR30 has been reported in various human malignancies, such as breast, ovarian and endometrial cancers, being associated with tumor growth and metastatic phenotype, and related to reduced survival rates in patients [6, 11, 12, 16]. The stimulation of GPR30 by 17β-estradiol (E2) can regulate cell survival, proliferation, migration, and invasion in estrogen-responsive tumors but is involved also in the development of resistance to conventional antiestrogens like tamoxifen [5, 6, 12, 16]. Therefore, the inhibition of GPR30 may provide a new promising target for endocrine therapy in estrogen-sensitive malignancies [5, 6, 11].

In the recent years, the flavone baicalein (Figure 1) was shown to efficiently intervene in E2-induced and GPR30-mediated signaling pathways in different breast tumor models, without affecting the GPR30 expression. In fact, baicalein significantly inhibited E2-elicited and GPR30-regulated transactivation of epidermal growth factor receptor (EGFR) as well as the downstream extracellular signal-regulated kinases 1/2 (ERK1/2) and Akt in human mammary non-tumorigenic epithelial cells MCF-10A and MCF-12A. The E2-enhanced levels of GPR30 target genes (e.g., *c-FOS*, *CTGF*, *CYR61*, *EGR1*) were also suppressed by baicalein treatment, showing that this natural flavone can be considered as a potential novel chemopreventive agent for reducing the risk of estrogen-dependent breast tumors [16]. Moreover, baicalein could suppress also the E2-induced GPR30 signal transduction in human breast cancer cells MCF-7 and SK-BR-3, leading to a significant decrease in E2-promoted migratory, adhesive, and invasive properties of malignant cells. Baicalein presents therefore a promising candidate for the treatment of GPR30-positive breast cancer metastasis [5, 6] (Table 1). Since traditional ERα antagonists (tamoxifen) act as GPR30 agonists increasing tumor invasiveness, in the future, co-inhibiting both the ERα and GPR30 receptors may provide a rational therapeutic strategy for the management of estrogen-dependent breast cancers [6, 16].

## Future perspectives in targeting GPCRs by flavonoids in anticancer therapies

The data presented in this review article clearly show that flavonoids can exert important anticancer effects through acting on different types of GPCRs, highlighting these natural polyphenols as potential drug candidates for the treatment of human malignancies. There are approximately 800 distinct GPCR genes encoded by the human genome, constituting the largest family of druggable targets [23, 24]. About one third of all FDA-approved drugs indeed interact with one or more GPCRs, suggesting that this class of receptors possesses an enormous clinical relevance [23]. Considering that a significant number of GPCRs have still remained orphan to date with no identified endogenous ligands and physiological functions [25], it is highly probable that there will be many more therapeutic targets identified in the future [26].

**Figure 2:**
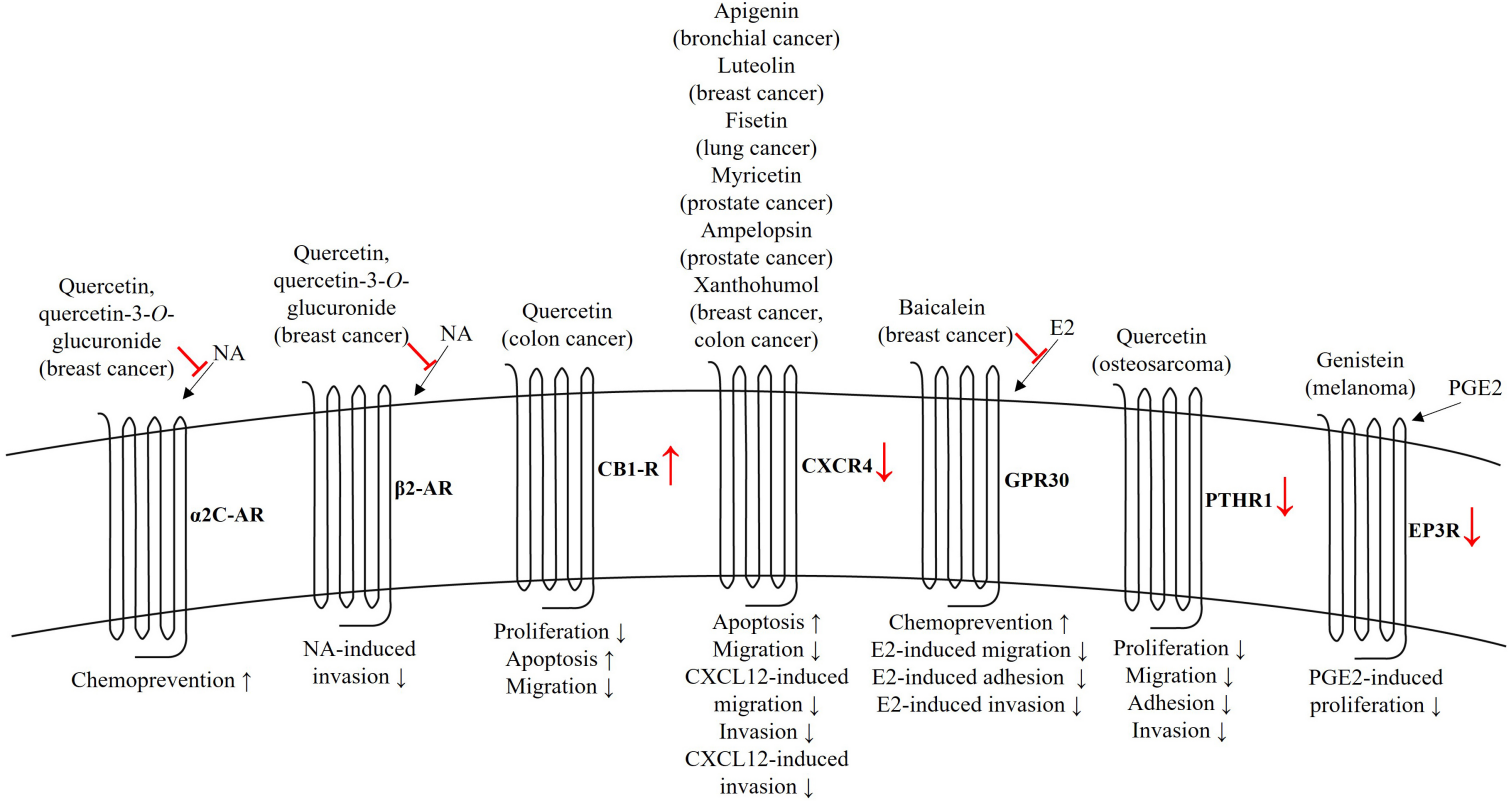
Anticancer effects of flavonoids mediated by GPCRs. The role of flavonoids is shown by red color. AR: adrenoceptor; CB_1_R: cannabinoid receptor 1; CXCL12: C-X-C motif chemokine ligand 12; CXCR4: C-X-C chemokine receptor type 4; E2: 17β-estradiol; EP3R: prostaglandin E receptor 3; NA: noradrenaline; PGE2: prostaglandin E2; PTHR1: parathyroid hormone receptor 1

The current knowledge about diverse anticancer activities of flavonoids mediated through regulating the expression and/or activity of GPCRs is summarized in Figure 2. In parallel to the increasing evidence about the involvement of various GPCRs in cancer initiation, development and progression, the findings of flavonoids on interfering with these processes have become more and more important. Therefore, further studies should unravel the precise molecular mechanisms occurring downstream the interactions between structurally different flavonoids and diverse types of GPCRs as well as their biological consequences. Also, the action of flavonoids on cross-talk between GPCRs and other cell membrane receptors, such as receptor tyrosine kinases and ion channels, is needed to be clarified to better understand the role of these phytochemicals in cancer and realize the possibilities to use these agents in combinatorial approaches with existing anticancer therapeutics. In addition, the pharmacological activity and selectivity of natural flavonoids may be further improved by diverse synthetic modifications. Although a great advantage of flavonoids lies in their relatively good safety profiles with only minimal side effects [27, 28], these compounds undergo a complex intestinal absorption and extensive bioconversion in the human body, revealing only poor bioavailability and low plasma concentrations. Indeed, the circulating levels of flavonoids following to their oral intake usually reach low micromolar doses, being, for example, 1.5 µM after consumption of 1 g quercetin per day for a period of four weeks [29] or 6.7 µM after single administration of 300 mg pure genistein in healthy human volunteers [30]. However, the pharmacokinetic parameters of flavonoids can be somewhat different between healthy people and cancer patients due to a number of metabolic changes accompanied by malignant processes [31]. Nevertheless, efficient drug delivery systems are also needed to be developed before these compounds can be clinically applied. All these efforts together would open new perspectives in identifying novel safe and more efficient anticancer therapeutic strategies for preventing and/or delaying the growth and metastasis of different human malignancies.

## Conclusions

Along with the unraveling of intracellular signaling networks driven by GPCRs in diverse malignant processes, intervention in these molecular events presents new options for the identification of novel and more efficient anticancer therapies. In this review article, the role of flavonoids in targeting these membranous receptors and regulating their downstream signaling pathways in cancerous systems is summarized, clearly showing that these plant-derived polyphenols may provide new molecular leads for finding novel agents to suppress GPCRs-mediated malignant processes. Further preclinical studies are highly needed to better understand the underlying molecular and pharmacological mechanisms, as well as to improve the activity and selectivity profiles of flavonoids in interacting with specific GPCRs.

## Abbreviations

4-OHE_2_: 4-hydroxyestradiol
Akt: protein kinase B
AR: adrenoceptor
Bax: Bcl-2-associated X protein
Bcl-2: B cell lymphoma-2
cAMP: cyclic adenosine monophosphate
CB1R: cannabinoid receptor 1
CXCL12: C-X-C motif chemokine ligand 12
CXCR4: C-X-C chemokine receptor type 4
E2: 17β-estradiol
ECM: extracellular matrix
EGFR: epidermal growth factor receptor
EMT: epithelial-mesenchymal transition
EP3R: prostaglandin E receptor 3
ERK1/2: extracellular signal-regulated kinases 1/2
GPCRs: G protein-coupled receptors
IL-8: interleukin-8
JNK: c-Jun N-terminal kinase
MAPKs: mitogen-activated protein kinases
MMP: matrix metalloproteinase
mTOR: mammalian target of rapamycin
NA: noradrenaline
NF-κB: nuclear factor-κB
PGE2: prostaglandin E2
PI3K: phosphoinositide 3-kinase
PIM1: Moloney murine leukemia virus 1
PKA: protein kinase A
PTH: parathyroid hormone
PTHR1: parathyroid hormone receptor 1
PTHrP: parathyroid hormone-related peptide
ROCKs: Rho-associated protein kinases
ROS: reactive oxygen species
STAT3: signal transducer and activator of transcription 3

## Declarations

### Copyright

© The Author(s) 2024.
